# Defective mitochondrial rRNA methyltransferase MRM2 causes *MELAS*-like clinical syndrome

**DOI:** 10.1093/hmg/ddx314

**Published:** 2017-08-25

**Authors:** Caterina Garone, Aaron R D’Souza, Cristina Dallabona, Tiziana Lodi, Pedro Rebelo-Guiomar, Joanna Rorbach, Maria Alice Donati, Elena Procopio, Martino Montomoli, Renzo Guerrini, Massimo Zeviani, Sarah E Calvo, Vamsi K Mootha, Salvatore DiMauro, Ileana Ferrero, Michal Minczuk

**Affiliations:** 1 Medical Research Council Mitochondrial Biology Unit, University of Cambridge, Cambridge CB2 0XY, UK; 2 Department of Neurology, Columbia University Medical Center, New York, NY 10032, USA; 3 Department of Chemistry, Life Sciences and Environmental Sustainability - University of Parma, Parma 43121, Italy; 4 Graduate Program in Areas of Basic and Applied Biology (GABBA), University of Porto, 4099-002, Portugal; 5 Metabolic Unit, A. Meyer Children's Hospital, Florence 50139, Italy; 6 Pediatric Neurology Unit and Laboratories, “A. Meyer” Children's Hospital, University of Florence, 50139, Italy; 7 Broad Institute of MIT & Harvard, Cambridge, MA 02142, USA; 8 Department of Molecular Biology and Howard Hughes Medical Institute, Massachusetts General Hospital, and Department of Systems Biology, Harvard Medical School, Boston, MA 02115, USA

## Abstract

Defects in nuclear-encoded proteins of the mitochondrial translation machinery cause early-onset and tissue-specific deficiency of one or more OXPHOS complexes. Here, we report a 7-year-old Italian boy with childhood-onset rapidly progressive encephalomyopathy and stroke-like episodes. Multiple OXPHOS defects and decreased mtDNA copy number (40%) were detected in muscle homogenate. Clinical features combined with low level of plasma citrulline were highly suggestive of mitochondrial encephalopathy, lactic acidosis and stroke-like episodes (MELAS) syndrome, however, the common m.3243 A > G mutation was excluded. Targeted exome sequencing of genes encoding the mitochondrial proteome identified a damaging mutation, c.567 G > A, affecting a highly conserved amino acid residue (p.Gly189Arg) of the MRM2 protein. *MRM2* has never before been linked to a human disease and encodes an enzyme responsible for 2’-*O*-methyl modification at position U1369 in the human mitochondrial 16S rRNA. We generated a knockout yeast model for the orthologous gene that showed a defect in respiration and the reduction of the 2’-*O*-methyl modification at the equivalent position (U2791) in the yeast mitochondrial 21S rRNA. Complementation with the *mrm2* allele carrying the equivalent yeast mutation failed to rescue the respiratory phenotype, which was instead completely rescued by expressing the wild-type allele. Our findings establish that defective MRM2 causes a MELAS-like phenotype, and suggests the genetic screening of the *MRM2* gene in patients with a m.3243 A > G negative MELAS-like presentation.

## Introduction

Mitochondria are eukaryotic intracellular organelles that play a central role in cellular metabolism by performing oxidative phosphorylation (OXPHOS) (1). Unique features of mitochondria are the presence of a 16.6 kb circular DNA molecule (mtDNA), encoding 22 tRNA, 2 rRNA and 13 protein subunits of the OXPHOS system, and intra-mitochondrial replication and translational machineries. Mitochondrial protein synthesis requires a number of structural and regulatory proteins, including mitoribosomal proteins, translation factors, aminoacyl tRNA synthetases, RNA processing enzymes, and other auxiliary factors. All of them are encoded by the nuclear DNA and translocated into mitochondria upon their synthesis in the cytoplasm (2).

Post-transcriptional chemical modifications of RNA, collectively referred to as epitranscriptome, are required for proper structure and function of RNA. Mitochondrial transcripts are also subject to enzymatic nucleotide modifications in human mitochondria. Systematic analysis of all bovine mitochondrial tRNAs (mt-tRNAs) reveled that approximately 7% of their residues undergo post-transcriptional modification, with the majority of these modifications being conserved in the human mitochondrial epitranscriptome (3,4). Also, a set of well-conserved chemical modifications to the small (12 S) and the large (16S) mitochondrial ribosomal RNAs (mt-rRNAs) has been identified and several proteins responsible for introducing these modifications have been characterized (5). In 12 S mt-rRNA, TFB1M is responsible for dimethylation of adenines A936 and A937 (6,7), whereas NSUN4 methylates cytidine at position 841 (8). The 16S mt-rRNA is also a substrate for one pseudouridylation (Psi1397), potentially introduced by RPUSD4 (9,10). Very recently identified modification, m^1^A947, has been shown to be introduced by TRMT61B (11). Finally, a group of closely related 2’-*O*-ribose methyltransferases, MRM1, MRM2 (FtsJ2) and MRM3 (RNMTL1) modify three nucleotide positions of the peptidyl transferase centre of 16S mt-rRNA, G1145, U1369 and G1370, respectively (12,13). Inactivation of the mt-rRNA modifiers often result in the inhibition of mitochondrial translation and/or mitoribosome biogenesis, however, the exact role of these RNA modifications is largely unknown.

Defects in mitochondrial translation due to problems in post-transcriptional mitochondrial (mt-) RNA nucleolytic processing, nucleotide modifications, stability or aminoacylation have been identified in patients with combined or isolated (complex I or IV) OXPHOS deficiencies and variable clinical phenotype. The central nervous system and heart are the most affected tissues with the majority of patients presenting with leukoencephalopathy, for example, TRNT1, TRIT1, NSUN3, TRMT5, DARS2, RARS2, EARS2, MTFMT (14–23) or hypertrophic cardiomyopathy for example, MTO1, ELAC2, GTPBP3, AARS2 (24–27). Neurological symptoms include developmental delay, ataxia, seizures, hypotonia, spasticity, and peripheral neuropathy. Hematological, hepatic and renal involvement has also been described in isolation and in multisystemic clinical syndromes, for example, TRMU, PUS1, YARS2, SARS2, HARS2, LARS2 (28–32).

Here, we report for the first time that defects in the nucleotide modification of mitochondrial ribosomal rRNA can lead to human disorders of mitochondrial respiration. We describe homozygous mutations in *MRM2* gene encoding the mitochondrial 16S rRNA methyltransferase MRM2 in a patient with MELAS-like encephalomyopathy, multiple OXPHOS deficiency and reduction of mtDNA copy number.

## Results

### Subject

**Figure 1.**
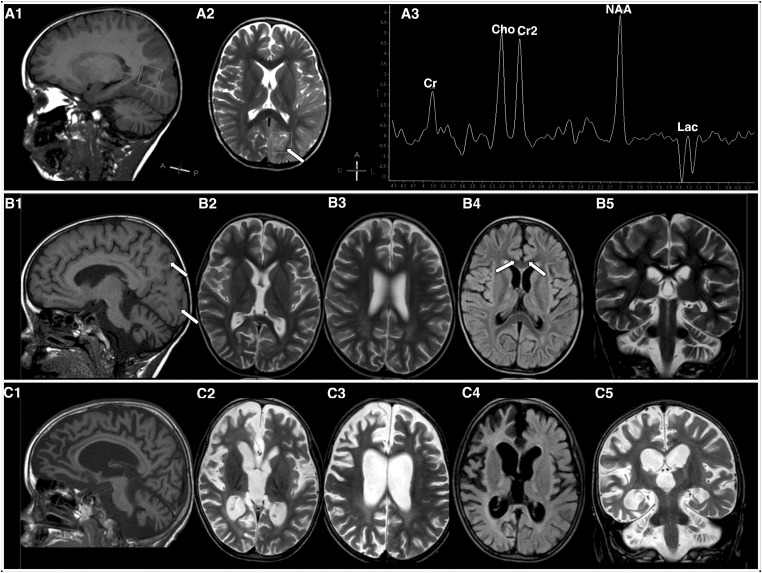
MRI brain imaging. (**A**) MRI abnormalities in the acute stage (48 h from status epilepticus). A1: Sagittal T2 weighted image showing initial signs of cerebellar atrophy. A2: Axial T2-weighted image showing hyperintensities in the left parieto-temporo-occipital area. A3: Spectroscopy analysis with ROI in the acute lesion showing reduction of NAA/CR, increase of Cho/CR and lactate peak. (**B**) Follow-up MRI (15 days): sagittal (B1) and coronal (B2) axial T2 (B2 and B3), FLAIR (B4) and coronal T2 (B5) T2-weighted images showing additional bilateral frontal lesions and cerebral and cerebellar atrophy. (C) MRI abnormalities in the chronic stage (one year after acute onset): Sagittal T1 (C1), axial T2 (C2 and C3), FLAIR (C4) and coronal T2 (C5) images showing severe progression of cerebral and cerebellar atrophy.

A 7-year-old boy born at term after uneventful pregnancy to non-consanguineous Italian parents presented at 8 months of life with developmental delay and a complex movement disorder characterized by generalized dyskinesia, featuring chorea, ballismus, also involving the cervical and oropharyngeal muscles, not responsive to levodopa and carbidopa treatment. Cerebrospinal fluid neurotransmitters and brain MRI (18 months of age) were unrevealing. The clinical course was stable in the first stage, but no motor or language milestones were acquired. At 4 years of age, the boy exhibited an acute deterioration during a febrile respiratory infection. He was admitted to pediatric intensive care for recurrent episodes of convulsive status epilepticus which were associated with multiple stroke lesions on brain MRI. Epilepsy proved refractory to combined anticonvulsant treatment and further complicated by mixed acidosis. He required induction of pharmacological coma, tracheostomy and nasogastric tube feeding. Metabolic workup including plasma aminoacids, acylcarnitine and lactate, urinary organic acids and orotic acid was unrevealing except for low levels of citrulline (9 μmol/l; n.r.17-53). Brain MRI on the 2nd day after the first episode of status epilepticus showed a focal lesion in the left parieto-temporal- occipital region with a ring of edema and restriction of diffusion. Cerebellar atrophy was also present. Magnetic resonance spectroscopy (MRs) with ROI (Region Of Interest) within the lesion showed a peak of lactate and reduction of NAA/Cr (Fig. 1A). Follow-up MRI (on day 10) confirmed the stroke-like lesion in the parieto-occipital region and revealed additional lesions in the right parsagittal frontal and fronto-insular cortex (Fig. 1B). MRs showed normalization of the lactate peak. The patient was able to overcome the acute episode, however, his neurological conditions were severely compromised with spastic quadriparesis, a complex dyskinetic movement disorder and *epilepsia partialis continua* resistant to anticonvulsant polytherapies. Repeat brain MRI at 1 year of follow-up showed severe cerebral and cerebellar atrophy (Fig. 1C). The clinical course was complicated by recurrent episodes of liver failure, hyperammonemia and rhabdomyolysis triggered by infections. He died at 7 years of age when a febrile illness progressed to sepsis. Brain MRI imaging, together with the clinical picture and the low level of plasma citrulline, was suggestive of MELAS. However, the common m.3243 A > G mutation in the mt-tRNA^Leu^ was excluded. Mitochondrial respiratory chain activities on muscle homogenate revealed multiple OXPHOS defects with Complex I and IV being severely affected while other complexes were at borderline level (Table 1). Quantitative PCR with DNA extracted from muscle revealed 40% of residual mtDNA copy number.

**Table 1.** Mitochondrial Respiratory chain activities in muscle homogenate

Enzyme normalized to CS	Patient	Normal range
Cytochrome c oxidase (IV)	**48**	120–220
Succinate-cytochrome c reductase (II+III)	17.8	15–28
DBH2-cytochrome c reductase (III)	61	60–100
NADH-CoQ_1_ reductase (I)	**6.7**	13–24
Succinate dehydrogenase (II)	10.8	10.7–17.4
Citrate synthase (nmol/min mg)(CS)	105	80–210

Defective values are highlighted in bold.

### Target exome sequencing revealed defect in *MRM2* gene

**Figure 2.**
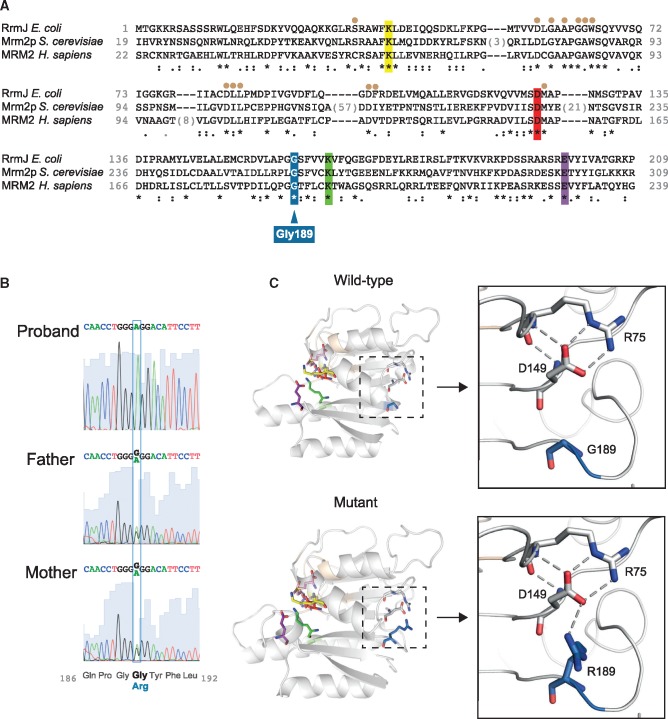
Detection of the p.Gly189Arg *MRM2* variant and its implications on protein structure. (**A**) Sequence alignment of MRM2 with homologs in *E. coli* and yeast *S. cerevisiae*. Conserved residues important for catalysis are coloured - yellow (Lys59), red (Asp154), green (Lys194) and purple (Glu229) (34). The mutation p.Gly189Arg is in blue. Brown dots above the sequence identify residues involved in SAM-binding. (**B**) Sanger sequencing results confirming segregation of the p.Gly189Arg variant. The c.567G>A mutation site is indicated by a blue frame. Protein sequence of the analyzed DNA fragment given below the chromatograms. GGG – Gly codon, AGG – Arg codon. (**C**) Modeling of the three-dimensional structure of human MRM2 with the p.Gly189Arg amino acid substitution. (i) Models of wild-type MRM2 and the p.Gly189Arg mutant with SAM in the active site were generated on the template of crystal structure (PDB ID 2NYU). The magnified regions show potential new amino acid interactions in the mutant protein (residue numbers are based on the human MRM2 sequence). SAM is colored pink.

A homozygous missense variant Chr7: 2274933 C > T (NM_013393: c.567 G > A) in the *MRM2* gene (encoding mitochondrial rRNA methyltransferase 2) has been identified. This variant has never been observed in the ExAC database and affects amino acid p.Gly189Arg (NP_037525). The Gly189 residue is extremely highly conserved across evolution, and is identical in 41/44 aligned vertebrate species as well as in yeast *S. cerevisiae* (Mrm2p) and even *E. coli* (RrmJ) (Fig. 2A). The variant was predicted deleterious by Polyphen algorithm (http://genetics.bwh.harvard.edu/pph/). Analysis of the run of homozygosity (ROH) for the MRM2: p.G189R homozygous variant showed a ROH of at least 16 kb in length. Sanger sequencing confirmed the mutation to be homozygous in the proband’s DNA and heterozygous in both parents’ DNA (Fig. 2B).

In the human MRM2 structure (PDB ID: 2NYU) Gly189 is not located in the vicinity of the site involved in *S*-adenosyl-methionine (SAM) binding and catalysis (Fig. 2C). We have modeled the p.Gly189Arg mutation into the MRM2 tridimensional structure using the wild-type crystal structure as a template. Based on structural analysis, we predict that the mutation of Gly189 to an arginine results in a potential new interaction with Asp149 (Fig. 2C). In the wild-type protein Asp149 forms a bond with Arg75. Although the role of Asp149 and Arg75 in the function of MRM2 is unknown, the potential Arg189-Asp149 interaction might have knock-on effects on the global structure of the protein reducing its catalytic function.

### Functional studies confirmed pathogenicity of the mutation

Mitochondrial respiratory chain activities in fibroblasts cultured in typical media revealed increased activity of citrate synthase (CS) and succinate dehydrogenase (Complex II), both indicators of mitochondrial mass. Normal but borderline activity of Complex I (NADH-CoQ_1_ reductase), III (DBH2 Cytochrome C Reductase) and IV (Cytochrome oxidase), were detected when normalized to CS. Galactose-media stress inhibits mitochondrial respiration and consequently can alter mitochondrial mass by inducing mitophagy and causing depletion of mitochondrial mass in patient’s fibroblasts (33). Similarly to previous reported cases, a profound reduction of mitochondrial mass with accompanied reduction of respiratory chain complex activities was identified when the patient fibroblasts were grown in media containing galactose as a sole carbon source (Supplementary Material, Fig. S1A). The *MRM2* gene was expressed normally in patient fibroblasts as demonstrated by analyzing mRNA level (by qRT-PCR) and steady-state level of the MRM2 protein (by Western blot immunovisualization) (Supplementary Material, Fig. S1B and C).

**Figure 3.**
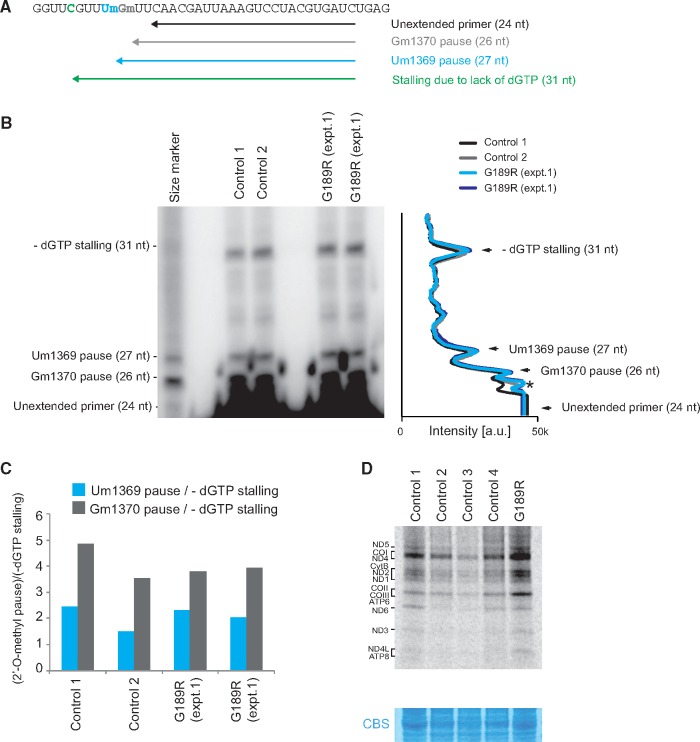
Studies of 16S mt-rRNA modification and mitochondrial translation in patient fibroblasts. (**A**) A radioactively-labeled, complementary primer (black arrow) is annealed to the large 16S rRNA and subjected to a reverse transcriptase primer extension (RT-PEx) reaction. The presence of Um1369 (modified by MRM2, blue arrow) and Gm1370 (modified by MRM3 (13) - grey arrow) results in RT-PEx pausing, producing shorter products. Reduced levels of Um1369 and Gm1370, however, lead to the extension reaction progress until stalling due to the lack of a dGTP (green), producing a longer product. (**B**) Representative PAGE separation and detection of RT-PEx products preformed on RNA extracted from control and patient fibroblasts (G189R), with a densitometric analysis presented to the right. (**C**) Quantification values representing the ratio of the intensity of the RT-PEx product specific for 2’-*O*-methylation (Um1369 or Gm1370) to the intensity of the dGTP-induced stalling. (**D**) Metabolic labelling of mitochondrial translation products with [^35^S]-methionine in control and patient fibroblasts (G189R). CBS: Coomassie blue stained gel as loading control.

It has been previously shown that the MRM2 methyltransferase introduces 2’-*O*-methylation at the U1369 (Um1369) of mitochondrial 16S rRNA and that its inactivation leads to a reduction of the intra-mitochondrial translation (12,13). Therefore, we set out to determine whether the *MRM2* variant identified in the patient results in diminished levels of Um1369 in 16S mt-rRNA and may impair mitochondrial translation. The detection of Um in RNA can be achieved by reverse transcription primer extension (RT-PEx). This approach relies upon pausing of the reverse transcriptase at a 2’-*O*-methylation site (13,18). To normalize RNA input, RT-PEx was performed in the absence of dGTP to cause stalling shortly downstream from the modification site (Fig. 3A). RT-PEx of a radioactively labeled primer annealing downstream of Um1369 using total RNA did not demonstrate any detectable changes in pausing at Um1369 in the patient sample as compared to the control samples (Fig. 3B and C). In further experiments, we investigated the mtDNA-encoded OXPHOS proteins by analyzing the steady state levels by western blotting and metabolic labeling using assay [^35 ^S]-methionine. We did not observe any detectable changes in mitochondrial translation (Fig. 3D and Supplementary Material, Fig. S1D). Taken together, our data suggest that patient-derived fibroblast cells did not show the disease phenotype, consistent with this tissue not being affected.

**Figure 4.**
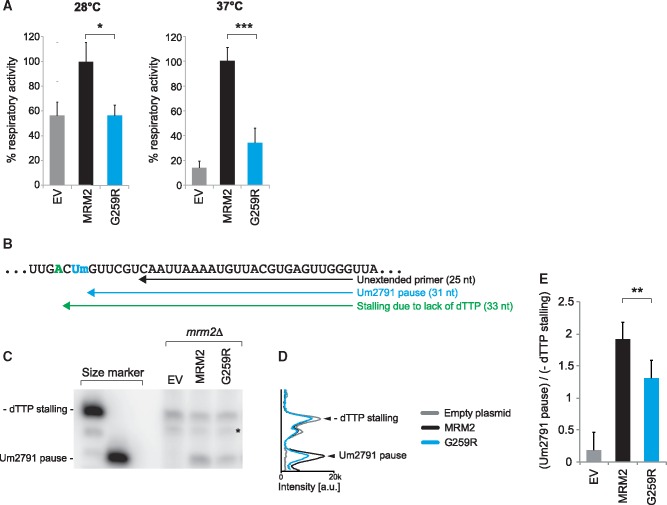
Causal role of the p.Gly189Arg *MRM2* variant in oxidative metabolism deficiency and mitochondrial rRNA modification. (**A**) Complementation assay in yeast. Yeast *MRM2* was cloned under its natural promoter upon PCR-amplification. PCR-based mutagenesis was performed to obtain the *mrm2^G259R^* mutant allele. The *MRM2* and *mrm2^G259R^*alleles cloned into the monocopy pFL38 vector were introduced into a strain harboring a disruption of genomic *MRM2* gene (*mrm2Δ*). EV denotes the empty pFL38 vector control. Oxygen consumption rate was recorded on intact cells grown at 28 °C and 37 °C in synthetic complete medium without uracil, supplemented with 0.6% glucose. Values were normalized to the rate of oxygen consumption of the *MRM2* transformant and represented as the mean of three independent experiments. Error bars = 1 SD, *P<0.05, ***P<0.001 with paired Student’s *t*-test. (**B**) A radioactively-labeled, complementary primer (black arrow) is annealed to the large rRNA and subjected to a reverse transcriptase primer extension (RT-PEx) reaction. The presence of Um2791 results in RT-PEx pausing producing a shorter product (blue). In the absence of Um2791, however, the extension progresses until stalling due to the lack of a dTTP (green), producing a longer product. (**C**) PAGE separation and detection of RT-PEx products preformed on RNA extracted from the *mrm2Δ* yeast transformed with pFL38 (EV), *MRM2* or *mrm2^G259R^*. (**D**) Densitometric analysis of the RT-PEx experiment shown in (C). (**E**) Quantification values representing the ratio of the intensity of the RT-PEx product specific for Um2791 to the intensity of the dTTP-induced stalling. Error bars = 1 SD; *n = *4, **P<0.01, with unpaired, two-tailed Student’s *t*-Test using the MRM2 sample.

In order to prove the pathogenicity of the mutation, we modeled the identified gene defect in yeast *S. cerevisiae*. The sequences of yeast and human MRM2 proteins are well conserved (Fig. 2A) and modify the corresponding residues in the large mitochondrial rRNA (13,34). We analyzed the yeast Mrm2p p.Gly259Arg substitution corresponding to the human p.Gly189Arg variant. Previous data indicated that the Mrm2p-null strain (*mrm2Δ*) shows thermosensitive respiratory deficiency (34). We have analyzed the *mrm2Δ* strain and confirmed this phenotype, detecting that the respiration is also affected in the permissive temperature (Fig. 4A). We rescued the respiratory defect by expression of a wild-type yeast *MRM2* allele (Fig. 4A). However, expression of the allele carrying the p.Gly259Arg mutation did not lead to a full recovery of the decreased mitochondrial respiratory activity (Fig. 4A). Next, we analyzed if the yeast Mrm2p p.Gly259Arg variant results in diminished Um2791 modification in yeast mitochondrial 21S rRNA that corresponds to Um1369 in human 16S mt-rRNA (13). We have adapted the RT-PEx approach as described above by using a primer that annealed downstream from the Um2791 site of 21S mt-rRNA (Fig. 4B). Performing the reaction without dTTP allowed for normalizing the relative pausing on Um2791 to the RNA input. RT-PEx reactions performed on RNA samples from the *mrm2Δ* strain demonstrated very low 2’-*O*-methyl pausing at U2791, consistent with the lack of the modification of this residue in the absence of Mrm2p (Fig. 4C–E). Expression of a wild-type *MRM2* allele restored the Um2791 modification, whereas expression of the allele carrying the p.Gly259Arg mutation did not result in similar detectable levels of Um2791 (Fig. 4C–E). In conclusion, these data are in agreement with a causal role for the p.Gly259Arg *MRM2* variant in the aberrant mt-rRNA modification and oxidative metabolism deficiency in the patient.

## Discussion

Stroke-like episodes have been recognized as a distinctive feature of maternally-inherited mitochondrial syndrome, MELAS (mitochondrial encephalopathy with lactic acidosis and stroke-like episodes), a multi-organ disease presenting with lactic acidemia and stroke-like episodes before age of 40 years, epilepsy, dementia and mitochondrial myopathy (35). The disease is due to mtDNA mutations in tRNA genes involved in the organelle protein translation with the most common variant m.3243 A > G in the mt-tRNA^Leu^. Energy depletion as well as NO (nitric oxide) deficiency are responsible for the pathogenesis of the disease as demonstrated by low plasma levels of an NO donor, citrulline, and the potential therapeutic effect of citrulline and its metabolite, arginine (36). Stroke-like episodes have been sporadically described as additional neuroradiological and clinical features in other mitochondrial disorders such as Alpers disease (37), coenzyme Q10 deficiency (38), 3-methylcrotonyl-CoA carboxylase 1 deficiency (39) and Leigh Syndrome French Canadian syndrome due to *LRPRRC* gene defect (40).

Here, we report a patient presenting with encephalopathy, stroke-like episodes, lactic acidosis, hypocitrullinemia, and multiple OXPHOS defects that are all clinical, biochemical and metabolic hallmarks for a diagnosis of MELAS-like syndrome. Although previously reported in isolated cases of MELAS syndrome (41–47), the complex movement disorders with chorea and ballism and the rapidly progressive clinical course with rhabdomyolysis and multi-organs failure represent distinctive clinical features in our case. Next generation sequencing with MitoExome sequencing excluded variants in mtDNA and identified a damaging homozygous mutation in a nuclear-encoded protein MRM2 playing a role in the mitochondrial protein translation machinery. Human MRM2 has been described as a putative uridine 2’-*O*-methyltransferase for the U1369 position of the mitochondrial 16S rRNA. Um1369 is located in the peptidyl transferase center and is implicated in the interaction of the ribosome with an aminoacyl(A)-site tRNA. In addition, proteomic studies have demonstrated the role of MRM2 in the assembly and/or stability of the large 39 S mitoribosomal subunit. The central role of MRM2 in the mitochondrial protein synthesis is confirmed by the multiple OXPHOS defects and reduction of mitochondrial translation and oxygen consumption rate in siRNA knockdown cell lines (13). Primary fibroblasts derived from the patient did not recapitulate the mitochondrial phenotypes observed upon MRM2 RNAi. This might be related to remarkable tissue-specificity observed in mitochondrial translational defects (18) and other mitochondrial diseases (48). Nonetheless, the yeast model showed the defect in cellular respiration and in modification of the equivalent uridine, Um2971, of yeast mitochondrial 21S rRNA. Complementation with wild-type *MRM2* allele rescued both of these defects, however, they were still present when we expressed the *MRM2* variant harboring the yeast mutation corresponding to the variant detected in the patient, confirming the pathogenicity of the mutation (Fig. 4).

Defective mitochondrial translation, caused by mutations in either the mitochondrial or nuclear genomes, is associated with a diverse group of human disorders characterized by impaired mitochondrial respiration. Within this group, an increasing number of mutations have been identified in genes involved in precursor mt-RNA processing (25,49,50) and in mt-tRNA epitranscriptome shaping (14,16–18,24,26,28,51). Most of them have been found in pediatric patients with combined respiratory chain activity defects by large gene screening with next generation exome sequencing (52,53). Clinical syndromes associated with defects in mtRNA metabolism are characterized by the variable combination of encephalopathy, myopathy, sideroblastic anemia, cardiomyopathy, renal or liver dysfunction. The central and peripheral nervous systems are predominantly affected with a generalized neuronal and/or myelin degenerative process (TRNT1, TRIT1, NSUN3 and TRMT5) or with selective damage of basal ganglia (GTPBP3) (54,55).

Here, we report for the first time, a MELAS-like clinical syndrome due to a nuclear-encoded protein, MRM2, further expanding the clinical and genetic heterogeneity of mitochondrial disorders and suggesting screening of MRM2 in patients with clinical and biochemical characteristics of MELAS syndrome with unknown genetic cause. Interestingly, a reduction in mtDNA copy number has been identified in muscle homogenate of our patient. A 40% residual mtDNA level is considered above the defined threshold for clinically-manifest mtDNA depletion (30%), but led us to hypothesize a secondary effect of the mutation on mtDNA replication. However, further studies are needed to confirm this hypothesis and explore a further, potential pathogenetic role in mitochondrial diseases associated with impaired mtDNA maintenance.

In conclusion, *MRM2* is a new disease-causing gene in mtDNA metabolism pathway that should be considered in the differential diagnosis of childhood-onset *epilepsia partialis continua* and stroke-like episodes.

## Materials and Methods

### Targeted exome sequencing

With the approval of the Pediatric Ethics Committees from the Tuscany Region and the informed consent of the parents of the affected individual, we performed targeted next-generation exome sequencing using the patient’s DNA in order to identify the genetic basis of the disease. An in-solution hybridization capture method (56) was used to target the MitoExome, corresponding to nuclear exons encoding known or predicted mitochondrial proteins as well as the mitochondrial DNA (mtDNA), which was then sequenced on the Illumina GA-II platform as previously described (52)*.* The *MRM2* variant was confirmed by direct Sanger sequencing exon 2 with the following primers: FWD: 5’-gtgattctgagcgacatggc-3’; REV: 5’-atgactctttcctgctggct-3’ and standard PCR amplification with 30 cycle at Tm 59 °C.

### Functional studies in patient-derived fibroblast cell line

Skin fibroblasts from the patient and controls were grown in basal condition with high-rich glucose Dulbecco’s Modified Eagle medium (DMEM) supplemented with 10% fetal bovine serum (FBS), 0.02% fungizone, and 1% penicillin-streptomycin or, under stressing conditions, with RPMI 1640 glucose-free medium with 10% regular FBS, 25 mM HEPES, 1.5 mM GlutaMAX™, 25 mM galactose, 0.02% fungizone, and 1% penicillin-streptomycin.

### Mitochondrial respiratory chain enzyme activities

Measurements were performed in cell lysate as previously described (57).

### MRM2 mRNA expression

RNA was extracted using PureLink RNA Mini Kit (Ambion, Life Technologies), treated with RNase-free DNase (Roche). cDNA was obtained with the VILO RT-PCR kit (Invitrogen). Quantitative reverse transcription (qRT-PCR) was performed by standard curve method, using TaqMan® Assays probes for *MRM2* (Hs00203647_m1) and glyceraldehyde-3-phosphate dehydrogenase (*GAPDH*) (Hs99999905_m1) as a standard loading control (Applied-Biosystem, Invitrogen).

### MRM2 steady-state protein level

40 μg of total protein was analyzed by electrophoresis in an SDS 12%-polyacrylamide gel, transferred onto PVDF membrane and probed with anti-MRM2 (1: 2000, ab60068, Abcam) and anti-vinculin (1: 5000, Sigma) antibodies. Peroxidase-conjugated anti-mouse IgG secondary antibodies (Santa Cruz Biotechnology) were used at a dilution of 1: 2000 and 1: 5000, for anti-MRM2 and anti-vinculin antibody, respectively. The protein bands were visualized by chemiluminescence (ECL, GE Healthcare).

### ^35 ^S-methionine metabolic labelling

To analyze translation of mitochondrially encoded proteins metabolic labelling was performed as previously described (13).

### MRM2 3 D model

The crystal structure of human MRM2 was obtained from the Protein Data Bank (PDB ID: 2NYU). To build the structure of MRM2 harboring the p.Gly189Arg amino acid substitution, both wild-type and mutated models were generated by SWISS-MODEL (58), using sequence inputs based on the protein reference sequence for MRM2 (UniProtKB: Q9UI43) (59), and 2NYU as a structural template. Since there is no spatial information for the 50 N-terminal residues of MRM2 in the 2NYU crystal structure, these were omitted in the input sequences. Global quality of the generated models was assessed by the QMEAN Z-score, which was 1.07 for the wild-type model and 0.77 for the p.Gly189Arg model. PyMOL (Molecular Graphics System, Version 1.8 Schrödinger, LLC) was used to visualize and render images from all structures.

### Heterologous MRM2 modeling in yeast

The yeast strains used were derived from BY4741*mrm2Δ* (MATa; *his3Δ1 leu2Δ0 met15Δ0 ura3Δ0 mrm2*:: KanMX4) (EUROSCARF collection). *MRM2* was cloned under its natural promoter by PCR-amplification and inserted into the pFL38 centromeric vector. The *mrm2^G259R^* mutant allele was obtained by PCR overlap technique with appropriate primers and cloned into the pFL38 vector. The pFL38 plasmid, empty or carrying *MRM2* or *mrm2^G259R^* alleles, were introduced into the BY4741*mrm2Δ* strain. Respiration rate was measured at 30 °C from yeast cell suspensions cultured for 18 h at 28 °C or for 16 h at 37 °C in SC medium supplemented with 0.6% glucose until exhaustion, using a Clark-type oxygen electrode (Oxygraph System Hansatech Instruments England) with 1 ml of air-saturated respiration buffer (0.1 M phthalate–KOH, pH 5.0), 0.5% glucose. For RNA extraction, cells were suspended in extraction buffer (0.6 M sorbitol, 10 mM imidazole, 0.5 mM EDTA, 0.1% BSA and 1 mM PMSF), were broken by vortexing on ice using glass beads and mitochondria were obtained by centrifugation. Then total RNA was prepared by extraction with hot acidic phenol (60). RNA used for RT-PEx study was extracted from an enriched mitochondrial fraction. All experiments, except transformation, were performed in synthetic complete (SC) medium (0.69% YNB without amino acids powder, ForMedium) supplemented with 1 g/l dropout mix without uracil (61).

## Supplementary Material

Supplementary Material is available at *HMG* online.

## Supplementary Material

Supplementary Figure S1
