# Supplementary material for: Defective mitochondrial rRNA methyltransferase MRM2 causes MELAS-like clinical syndrome
Source: Hum Mol Genet. 2017 Aug 25;26(21):4257–66. doi: 10.1093/hmg/ddx314 (PMC5886288; doi:10.1093/hmg/ddx314)
Supplement: Supplementary Figure S1 [file suppl_info_ddx314.docx]

**Supplementary Information**

**Defective mitochondrial rRNA methyltransferase MRM2 causes *MELAS*-like clinical syndrome**

Garone *et al.*

**Figure S1 | Functional studies in skin-derived patient fibroblast cell line**

**(A)** Mitochondrial respiratory chain activities measured in the patient fibroblasts (G189R) grown in galactose-containing medium. Mitochondrial mass measured by assessing the levels of CS and SDH. CS= citrate synthase; I+III=NADH-cytochrome c reductase; II+III= Succinate cytochrome c reductase; SDH= succinate dehydrogenase; IV= cytochrome c oxidase

**(B-C)** Steady-state level of the MRM2 mRNA (B) and protein (C) analyzed by qRT-PCR, and western blotting, respectively, in the patient –derived fibroblasts (G189R).

**(D)** Steady-state level of OXPHOS proteins analysed by western blotting in the MRM2 patient fibroblasts (G189R).
